# Points from Hospital Reports

**Published:** 1924-06

**Authors:** 


### POINTS FROM HOSPITAL REPORTS.

### West Suffolk General Hospital, Bury St. Edmunds.

Increased work, reduced expenditure, and a balance of £2,256 on the right side at the end of the year are ample justification for describing 1923 as a successful one for this institution of 100 beds. The Employers' and Workers Fund Contributory Scheme has made very satisfactory progress during the year both in the town section and the country section. The result—£1,240 from the former and £2,133 from the latter, total £3,373—speaks for itself, especially when we state that the total expenditure is little more than £10,000. Without making an invidious distinction—there are too many indefatigable workers labouring in the good cause for that—we must express our concurrence with the committee in their statement that the hospital is most fortunate in having such a splendid worker as Miss Holden, who is not only the chief organiser of the country organisation of the contributory scheme, but also of the egg collection associated with it. Ninety-one villages participate in this egg offering, the total number of eggs reaching the huge figure of 33,291! It is very pleasant to read the committee's appreciation of the work of their secretary, Miss Hardwicke. Our surprise is therefore the greater that the amount of "official salaries" is the very modest sum of £123 12s. 1d. This interesting report would be improved by a good index.

### Edinburgh Royal Infirmary.

This report for the year ended September 30, 1923, records a very full and successful year's work. The number of patients treated was higher, the total income more than sufficient to meet the expenditure of the year and the expenditure fell. Points of outstanding interest are the receipt of the remarkable number of fifty-three legacies of the total value, in round figures, of £51,000, and the very serious continued increase in the waiting list, which, on New Year's Day, reached the portentous figure of 1,787. This circumstance has led the managers to call the attention of the contributors and the community generally to the immediate and pressing need for the further expansion of the infirmary, already the largest voluntary hospital in the kingdom. As it is undesirable to increase the buildings on a site already congested, expansion involves obtaining possession of additional land, and the Committee of Contributors express the hope that the managers will be able to arrange for the acquisition of an extensive site, conveniently situated and well suited for the purpose, at an early date. A great undertaking, therefore, confronts the managers of this renowned hospital.

### Royal West Sussex Hospital, Chichester.

The year 1923 was, on the whole, an uneventful one for the West Sussex Hospital, but the record contained in the annual report is a very good one for all that. An excellent year's work was done, the income was fairly maintained and economical administration effected appreciable decreases in expenditure, with the result that there was a small balance to the good at the end of the year. This is very satisfactory, for, difficult as it is to raise funds, it is still more difficult and far more uncommon to spend them wisely. Nor is economical management always appreciated as it appears to be at Chichester. We read with much satisfaction in the closing paragraph of the report: "The Board wish to place on record their appreciation of the services of their secretary and for his labours on behalf of the hospital in many directions." We heartily congratulate Mr. Herbert S. Aylmore, not only on his achievement, but on having an appreciative Board.

### Chesterfield and North Derbyshire Royal Hospital.

The year 1923 was full of varied activities, the chief of which was the extension scheme, now completed, at a total cost of about £50,000, of which all but some £5,000 has been provided. A somewhat long waiting list is giving rise to the question whether the time is not approaching when the number of beds, now 168, will have to be increased. In this connection the Board observe: "The crux of the matter is that, while funds are available for the maintenance of more beds, the capital sum is not available." The obvious comment on this pronouncement is that it seldom is, but a "note" to the report to the effect that, since the end of the year, Mr. Charles P. Markham has made a gift of shares valued at about

£10,000 tends to suggest that the case of the Derbyshire Royal Hospital mayturn out to be the exception which proves the rule. We sincerely hope that it will.

BRADFORD ROYAL INFIRMARY.

The contrast of the financial position at the beginning and at the end of 1923 is a truly magnificent transformation scene, on which we congratulate not only the administration of the hospital, but the people of Bradford generally. In the year 1922 the expenditure exceeded the income by £3,301, and the year ended with an accumulated debit of £13,431. Last year the expenditure fell short of the income by £15,071, and, in addition, the accumulated debit was entirely cleared off. When we add that the work of the hospital was well maintained and the expenditure reduced during the year, all will agree that the Bradford Royal Infirmary is deservedly flourishing.

GRAVESEND HOSPITAL.

This report is a very interesting record of an excellent year's work. The hospital is very much alive, and it is specially pleasing to notice the unusually large proportion of its supporters who, not content, as is unfortunately so common, with giving a sum of money and concerning themselves no further in the affairs of the institution, are keen contributors of personal service, busying themselves most successfully in divers directions. The Ladies' Association is a striking example. Its income for the year was £937, and it is not surprising to learn that it is in a position not only to supply all the new linen the hospital requires, but, by the busy fingers of its numerous members, to maintain it in repair. We cannot find in the report the number of beds available in the hospital, although doubtless it is there: an index might help to discover the information. In seeking it we came across some excellent X-Ray photographs showing a needle in the hand, a bullet in the foot and a fractured fore-arm before and after operation. This in turn led to the revelation that the radiographer is the secretary, whereupon some little peculiarities in the report and accounts served rather to enhance our interest than to provoke criticism. With so much vitality among the hospital's adherents an excess of expenditure over income of £732 will not give the administration many sleepless nights.

